# Meta-Analysis of Functional Neuroimaging and Cognitive Control Studies in Schizophrenia: Preliminary Elucidation of a Core Dysfunctional Timing Network

**DOI:** 10.3389/fpsyg.2016.00192

**Published:** 2016-02-17

**Authors:** Irene Alústiza, Joaquim Radua, Anton Albajes-Eizagirre, Manuel Domínguez, Enrique Aubá, Felipe Ortuño

**Affiliations:** ^1^Department of Psychiatry and Clinical Psychology, Clínica Universidad de NavarraPamplona, Spain; ^2^Instituto de Investigación Sanitaria de NavarraNavarra, Spain; ^3^Department of Psychosis Studies, Institute of Psychiatry, Kings CollegeLondon, UK; ^4^FIDMAG Germanes Hospitalaries Hospital Sant RafaelBarcelona, Spain; ^5^Centro de Investigación Biomédicaen Redde Salud MentalBarcelona, Spain

**Keywords:** timing, cognition, neuroimaging studies, cognitive control, schizophrenia, SDM-meta-analysis

## Abstract

Timing and other cognitive processes demanding cognitive control become interlinked when there is an increase in the level of difficulty or effort required. Both functions are interrelated and share neuroanatomical bases. A previous meta-analysis of neuroimaging studies found that people with schizophrenia had significantly lower activation, relative to normal controls, of most right hemisphere regions of the time circuit. This finding suggests that a pattern of disconnectivity of this circuit, particularly in the supplementary motor area, is a trait of this mental disease. We hypothesize that a dysfunctional temporal/cognitive control network underlies both cognitive and psychiatric symptoms of schizophrenia and that timing dysfunction is at the root of the cognitive deficits observed. The goal of our study was to look, in schizophrenia patients, for brain structures activated both by execution of cognitive tasks requiring increased effort and by performance of time perception tasks. We conducted a signed differential mapping (SDM) meta-analysis of functional neuroimaging studies in schizophrenia patients assessing the brain response to increasing levels of cognitive difficulty. Then, we performed a multimodal meta-analysis to identify common brain regions in the findings of that SDM meta-analysis and our previously-published activation likelihood estimate (ALE) meta-analysis of neuroimaging of time perception in schizophrenia patients. The current study supports the hypothesis that there exists an overlap between neural structures engaged by both timing tasks and non-temporal cognitive tasks of escalating difficulty in schizophrenia. The implication is that a deficit in timing can be considered as a trait marker of the schizophrenia cognitive profile.

## Introduction

Temporal processing is central to many aspects of human cognition. Accurate judgment of elapsed time is associated with a broad range of activities from relatively basic tasks, such as planning or sequencing, to the higher order processes involved in driving a car, doing sport, playing music etc. According to Navon (1978), time occupies the highest level of the hierarchy of dimensions that forms our perception of the world. In view of the primacy of timing in human cognition, it has been suggested that timing dysfunction lies at the root of deficits (such as planning and aspects of decision making) observed in schizophrenia (Volz et al., 2001; Macar and Vidal, 2009).

That a temporal processing deficit exists in schizophrenia is increasingly recognized on the basis of phenomenological, clinical, and neurobiological observations. Although this deficit was described at the beginning of the last century, it continues to be of interest in current research.

Over the last decade, study of timing in schizophrenia has been fostered by two main factors. First, different models of schizophrenia pathogenesis implicate time perception. For example, Andreasen's theory of cognitive dysmetria (1999) conceptualizes schizophrenia as “*a misconnection in the fluid and coordinated sequences of thought and action stemming from a dysfunction of the cortico-cerebellar-thalamic-cortical circuit*.” Thus, this theory proposes that a disturbance in temporal coordination of information processing may underlie many symptoms of this mental disease (Davalos et al., 2011). Alternatively, Franck et al. (2005) maintained that schizophrenia is related to an excessive temporal integration of events, which leads to classic symptoms. To the degree that psychopathological dimensions of delusions, hallucinations and disorganized speech and behavior can be conceptualized as expressions of dysfunctional neural timing, findings related to such psychopathological dimension are relevant to our understanding of the pathophysiology of schizophrenia. From a phenomenological perspective, schizophrenia can be regarded as a structural breakdown of time consciousness (Vogeley and Kupke, 2007).

The second big reason for research into timing in schizophrenia stems from the idea that the real-life functional difficulties experienced by patients are better accounted for by timing impairment than by dysfunctions in executive control (Volz et al., 2001; Davalos et al., 2003). The potential impact of timing disturbance on cognition and daily behavior is great, and so knowledge of the etiology of timing deficits in schizophrenia may provide important insights into disease pathology.

Controversy remains, however, regarding the existence of a genuine timing disorder in schizophrenia. It is unclear whether disruptions in timing are due to primary disturbances in central temporal processes (perceptual or biological) or to secondary well-known disease-related cognitive impairments that include attention, declarative and working memory or executive functions. Regarding secondary disruptions, deficits associated with a cognitively controlled timing mechanism (for measuring duration in the order of seconds) would be expected to be different from deficits in an automatic mechanism (for measuring duration at the sub second scale). In fact, performance is found to be equally impaired for both duration ranges, and this suggests that the timing deficit in schizophrenia is essential and primary. The deficit seems to be independent of the length of duration that needs to be timed and also independent of more generalized cognitive impairments (Ciullo et al., 2015).

On the other hand, recent meta-analyses indicate that temporal processing is mediated by a cognitive task requirement (Radua et al., 2014a). Task requirements have an effect on the timing process engaged and on the neural substrate involved, not only in schizophrenia patients but also in normal human timing (Wiener et al., 2010).

Since temporal cognition is a fundamental “basic unit of ability” on which other cognitive and behavioral processes are based (Allman and Meck, 2012), complex cognitive functioning depends on underlying temporal constraints (von Steinbüchel and Pöppel, 1993). Accordingly, temporal processing plays a role in determining a wide range of cognitive processes.

According to the Scalar Expectancy Theory (SET), time processing involves multiple cognitive processes: an internal clock, short- and long- term memory, and decisional processes (Gibbon et al., 1984). In this sense, cognitive processes comprise allocation of attentional resources to the perception and encoding of incoming temporal information, storage and retrieval of the temporal percept in long-term memory, and comparison with other percepts in working memory (Piras et al., 2014). Alterations in any stage or aspect of the system are expected to result in individual and pathophysiological differences. Neuroimaging studies have focused on microanalysis of specific and independent brain networks related to each of the three SET subcomponents (Allman and Meck, 2012).

To the degree that timing is related to other cognitive domains such as attention and working memory, how timing is carried out and the neural networks responsible are relevant to our understanding of healthy cognition (Buhusi and Meck, 2005). The relationship has been proposed to stem from the involvement in timing not only of cortical structures such as the dorsolateral prefrontal cortex but also of other regions, such as the supplementary and pre-supplementary motor areas. The cortical structures are known to play a role in normal cognitive functions, and the motor areas have been identified as crucial for linking cognition to action (Basso et al., 2003). In view of the relationship, there has been increased interest from researchers of schizophrenia in the study of timing.

Timing and other cognitive processes are known to share brain networks (Gómez et al., 2014); some of the cognitive processes involved are attention, automatic or controlled behavior change, working memory, and the degree of concentration required depending on the level of difficulty of the task.

Functional magnetic resonance imaging (fMRI) is well suited to further investigate the nature of timing deficits in schizophrenia since it provides information about the task-related responses across the whole brain. To date there are only a few fMRI studies that examine timing in schizophrenia (Volz et al., 2001; Davalos et al., 2011), but findings suggest that timing deficits in schizophrenia might be due to a combined impairment of timing mechanisms in the basal ganglia or thalamus and impaired attentional or mnemonic resources organized in prefrontal cortices (Davalos et al., 2011).

Examination of timing in schizophrenia patients, who exhibit cognitive dysfunctions, can be regarded as a valid heuristic approach to explain the essence of the relationship between timing and cognition (i.e., whether the interrelation results from specific co-variation of common temporal processes or from coincidental co-variation in the cognitive components shared by the two functions; Piras et al., 2014).

Cognitive effort is an aspect of every cognitive process and refers to the level of difficulty of the cognitive task and to the consequent mental effort that individuals need to apply to achieve the cognitive aim (Radua et al., 2014a).

Daily tasks demand different levels of cognitive control, and therefore, continuous modulation of the level of effort is needed. Changes in cognitive load require the participation of common cerebral networks.

The neural mechanisms of timing are related to other cognitive functions; cognitive control and accurate executive functioning require the participation of functional and neuroanatomical components of time perception (Radua et al., 2014a). In the context of schizophrenia, neurobiological dysfunctions, or cognitive impairments have been demonstrated to interfere with certain levels of temporal processing, for example, in interval discrimination tasks (Roy et al., 2012).

Given the interrelation between timing and neuropsychological processes, and assuming that pathophysiological distortions in time can depend on and reflect neuropsychological deficits characteristic of neuropsychiatric disorders, the study of timing can be a way to research cognitive dysfunction. Impaired timing has been reported in diseases associated primarily with dopaminergic and fronto-striatal dysfunctions such as in schizophrenia. It has been suggested that the study of timing in schizophrenia can reveal important information on the core cognitive disturbances of this disorder (Matell and Meck, 2004; Wiener et al., 2011).

The study of timing is relevant to our understanding of neurobiological and cognitive abnormalities in schizophrenia. Brain lesion and neuroimaging studies have shown that the cortico-cerebellar-thalamic circuit engaged in temporal processing is involved, in terms of impaired activity coordination among the different brain regions, in the disease's pathophysiology (Andreasen et al., 1999). The cortico-cerebellar-thalamic network involves the bilateral pre-supplementary and supplementary motor area (SMA), the right middle frontal region, the right inferior parietal region, the insula, the left putamen, the right posterior cerebellum, the superior temporal gyrus, the right thalamus, the right middle frontal gyrus, and the left superior temporal gyrus (Volz et al., 2001; Ivry and Spencer, 2004; Ortuño et al., 2011). Hypothetically, cognitive deficits, which in turn lead to impaired timing, can be interpreted as being the result of a disturbance in the functioning of the cortico-striatal pathways; and this same disturbance contributes to a variety of other symptoms associated with schizophrenia (Ward et al., 2011).

We hypothesize that an impaired temporal/cognitive control network underlies the dysfunctional cognition of higher processes in schizophrenia. Our emerging hypothesis is that timing structures are activated either by increased demand on working memory; by the need to shift attention from lower, automatic, levels to higher, controlled, levels; or by certain complex mental operation tasks. Thus, a dysfunctional time estimation network may be linked with other critically impaired functions in schizophrenia.

In the current study, we seek to determine whether schizophrenia patients present a dysfunctional activity pattern in a cognitive control circuit and whether such a pattern matches the pattern involved in timing. To these two ends, we conducted a SDM meta-analysis of published neuroimaging data and then performed a multimodal analysis to identify common brain regions in the findings of that SDM meta-analysis and our previously published activation likelihood estimate (ALE) meta-analysis of neuroimaging of time perception in schizophrenia patients.

## Materials and methods

### Meta-analysis of cognitive difficulty

Two electronic bibliographic databases were searched (PubMed and Web of Science) to identify fMRI studies reporting brain activation patterns associated with changes in cognitive control and effort. This search was limited to literature published between January 2012 and December 2014. Based on preliminary searches, this timeframe was deemed to yield a sufficient number of studies for testing the hypothesis of the present work (please note that we did not intend to conduct an exhaustive meta-analysis). We compare the findings of our SDM meta-analysis to those obtained through our previously published ALE meta-analysis. The previous meta-analysis comprised only three studies, and so inclusion of a disparately large number of studies in the new meta-analysis was not a priority. Keywords were (fMRI) AND (attention OR working memory OR executive functions OR controlled processes) AND (schizophrenia).

Inclusion criteria were: (1) use of a standardized or experimental designed cognitive task; (2) samples composed of healthy volunteers and/or patients with schizophrenia; (3) availability of peak coordinates or statistical parametric maps, either in the published article or after contacting the authors; (4) use of whole brain analyses; (5) use of a constant threshold in the different regions of the brain.

Exclusion criteria were (1) studies from which peak coordinates or statistical parametric maps could not be retrieved from the published article or after contacting the authors; (2) studies whose analyses were limited to specific regions of interest; (3) studies in which different thresholds were used in different regions of the brain; (4) functional neuroimaging studies with techniques other than fMRI (e.g., PET, SPECT); (5) studies that did not specify at least two levels of difficulty of cognitive task or did not use levels with a clear difference in difficulty; (6) studies that considered a resting state or baseline as the lower level of difficulty; (7) studies based on *Independent Component Analysis* (ICA); (8) case reports, qualitative studies, reviews, and meta-analyses.

No language restrictions were imposed.

Two reviewers independently assessed the studies against the inclusion/exclusion criteria in a standardized manner. Keywords were initially screened in the title and abstract. Afterwards, the full text of eligible studies was analyzed. Any conflicts in reviewers' decisions about inclusion vs. exclusion were resolved through discussion between the two reviewers.

For each selected study, the following information was extracted: number of participants (patients and controls), cognitive tasks and contrasts (Table 1), and peak coordinates (MNI or Tailarach) and their effect size (t statistic, z score, *p*-value).

**Table 1:** **Studies of cognitive control included in our SDM meta-analysis**.

**Authors**	**Sample**	**Task**	**Included contrast**
1. Anticevic et al., 2012	28 SZ 24 HC	Simple perceptual decision task	Negative vs. Neutral distraction
2. Avsar et al., 2013	14 SZ 14 HC	Delay-discounting task	Delay-discounting vs. Sensorimotor control; Hard vs. Easy trial difficulty
3. Bender et al., 2013	14 SZ 13 HC	Volitional and visually guided saccades task	Simple volitional vs. Visually guided saccade
4. Bjorkquist and Herbener, 2013	14 SZ 14 HC	Social perception task	Social vs. Nonsocial images
5. Bleich-Cohen et al., 2014	16 SZ 20 HC	N-back WM task	2-back vs. 0-back
6. Das et al., 2012	20 SZ 19 HC	ToM task	ToM animation vs. Random animation
7. de la Fuente-Sandoval et al., 2012	12 SZ 13 HC	An experimental pain tolerance task	Painful vs. Non-painful thermal stimuli
8. Dowd and Barch, 2012	25 SZ 20 HC	Pavlovian reward prediction task	Money cue vs. No money cue
9. Eich et al., 2014	18 SZ 18 HC	An item-recognition task	Pre cue vs. Post cue; Lure vs. Control
10. Esslinger et al., 2012	27 SZ 27 HC	Monetary reward & face-matching tasks	Monetary vs. Control; Famous vs. Non-famous stimuli
11. Gradin et al., 2013	15 SZ 20 HC	Pavlovian reward learning task	Reward vs. No reward
12. Grillon et al., 2013	15 SZ 15 HC	Refresh task	Refresh vs. Read
13. Harvey and Lepage, 2014	28 SZ 26 HC	A social and nonsocial picture recognition memory task	Old social pictures vs. New social pictures; Old nonsocial vs. New nonsocial pictures
14. Hashimoto et al., 2014	17 SZ 17 HC	One-back visual task	Biological motion (BM) vs. Static state (ST); Scrambled motion (SM) vs. ST; BM vs. SM
15. Li et al., 2012	12 SZ 12 HC	Facial emotion processing task	Happy vs. Neutral; Fearful vs. Neutral
16. Kauppi et al., 2014	63 SZ 118 HC	WM N-back task	2-back vs. 0-back
17. Lakis and Mendrek, 2013	37 SZ 37 HC	An emotion processing task	Negative vs. Neutral; Positive vs. Neutral
18. Lee J. et al. (2014)	20 SZ 26 HC	A 4-Dot object substitution masking task	Stimulus-onset asynchrony (SOA)1 vs. SOA234
19. Lee J. S. et al., 2014 2014	15 SZ 16 HC	Facial expression task	Emotional vs. Meaningless
20. Lesh et al., 2013	43 SZ 54 HC	Stroop and AX-CPT	I vs. C; B vs. A
21. Lindner et al., 2014	36 SZ 40 HC	Facial processing task	Masked disgust vs. Neutral; Unmasked disgust vs. Neutral
22. Linnman et al., 2013	15 SZ 13 HC	A classical conditioning paradigm	Conditioned stimulus (CS)+end vs. CS-end; Unconditioned stimulus (US) vs. CS-end
23. Liu et al., 2014	15 SZ 15 HC	Referential task	Self vs. Other
24. Mashal et al., 2013	14 SZ 14 HC	Metaphor comprension task	Novel metaphors vs. Meaningless word pairs; Novel metaphors vs. Conventional metaphors; Novel metaphors vs. Literal expressions
25. Matsumoto et al., 2013	6 SZ 6 HC	Rorshach inkblots speech	Between clause vs. Within clause pauses
26. Matsuo et al., 2013	46 SZ 46 HC	Sternberg verbal WM task	High load vs. Low load
27. Natsubori et al., 2014	20 SZ 20 HC	Visual lexical decision task	Non-words vs. Words
28. Niendam et al., 2014	35 SZ 35 HC	Cue phase of AX-CPT	Cue B vs. Cue A
29. Pauly et al., 2013	13 SZ 13 HC	Self evaluation task	Other vs. Lexical; Self vs. Lexical; Self vs. Other
30. Pedersen et al., 2012	15 SZ 14 HC	“Moving shapes” paradigm	ToM vs. non ToM
31. Ragland et al., 2012	20 SZ 19 HC	WM task	Relational (reorder trials) processing vs. Item-specific (rehearse trials) processing
32. Sapara et al., 2014	18 SZ 20 HC	N-back WM task	1-back vs. 0-back; 2-back vs. 0-back; 2-back vs. 1-back
33. Shad et al., 2012	17 SZ 15 HC	Self-awareness task	Self-directed sentence-stimuli vs. Other-directed sentence-stimuli within the self-referential (SR) cue epoch; Self-directed sentence-stimuli vs. Other-directed sentence-stimuli within the other-referential cue epoch
34. Smieskova et al., 2012	21 SZ 20 HC	N-back WM task	2-back vs. 0-back
35. Straube et al., 2013	16 SZ 16 HC	Gesture processing task	Metaphoric vs. Iconic
36. Subramaniam et al., 2014	30 SZ 15 HC	N-back WM task	2-back vs. 0-back
37. Tully et al., 2014	23 SZ 24 HC	Multi-source interference task	Negative vs. Neutral; Neutral interferente vs. Neutral control; Negative interferente vs. Negative control
38. van der Meer et al., 2013	20 SZ 20 HC	An emotion regulation task	Reappraise vs. Attend negative
39. van der Meer et al., 2014	47 SZ 21 HC	A self-reflection task	Self vs. Semantic; Other vs. Semantic
40. Vercammen et al., 2012	20 SZ 23 HC	Verbal emotional go/ no-go task	Inhibit negative vs. Neutral; Inhibit positive vs. Neutral
41. Vercammen et al., 2013	18 SZ 22 HC	An emotional go/no-go task	Inhibit negative vs. Inhibit neutral
42. Villalta-Gil et al., 2013	22 SZ 31 HC	Facial emotion processing task	Emotions at 50% intensity vs. Neutral emotions; Fearful faces vs. Neutral emotions; Match emotion vs. match gender (neutral faces)
43. Villarreal et al., 2014	14 SZ 14 HC	Social functioning tasks	Theory of mind task-eyes (EToM) vs. Test of Adaptive Behavior in Schizophrenia (TABS)

*SZ, schizophrenic patients; HC, healthy controls; WM, working memory, ToM, Theory of Mind; CPT, Continuous Performance Task*.

Data were spatially summarized with anisotropic effect-size signed differential mapping software (ES-SDM, http://www.sdmproject.com; Radua and Mataix-Cols, 2009; Radua et al., 2011, 2014b), a novel quantitative voxel-based meta-analytic method. First, peak coordinates and their *t*-values were used to recreate an effect-size map of the BOLD response for each contrast. These maps included both activations (easy > difficult) and deactivations (difficult > easy; Radua and Mataix-Cols, 2010).

We applied multi-source pre-processing of the data in order to obtain more accurate and thorough recreations of the statistical tridimensional maps of the comparisons between patients and controls for the difficult vs. easy contrast. For each study, we used signed differential mapping (SDM) and the reported peak coordinates and *t*-values to separately recreate:

1. the map of the difficult-easy contrast in patients (we will refer to this map as the *patients-only map*), where values were positive for activations (difficult > easy) and negative for deactivations (difficult < easy);
2. the map of the same contrast in healthy controls (we will refer to this map as the *controls-only map*); and
3. the map of the comparison between patients and controls in this contrast (we will refer to this map as the *combined map*), where values were positive for hyperactivations (patients > controls in difficult > easy) or for failures of deactivation (patients < controls in difficult < easy), and negative for hypoactivations (patients < controls in difficult > easy) or hyperdeactivations (patients > controls in difficult < easy).

Results of the pre-processing were inspected to ensure that the recreated maps coincided reasonably well with the results reported in the studies. When two or more contrasts involved overlapping samples, they were combined into a single average map with decreased variance (Rubia et al., 2014; Alegria et al., Submitted).

Next, controls-only maps were subtracted from patients-only maps to obtain *subtraction maps*. This calculation took into account that the maps were not means but *t*-values:

$$\begin{array}{l} t_{Patients-Controls}=\sqrt{\frac{n_{Controls}}{N}}\cdot t_{Patients}-\sqrt{\frac{n_{Patients}}{N}}\cdot t_{Controls} \end{array}$$

Note that the recreation of tridimensional maps from peak information requires that the combined maps contain more accurate information in voxels close to the peaks of the differences between groups. Conversely, the subtraction maps have accurate information in voxels close to peaks of activation or deactivation in one or both groups. Thus, a more accurate map can be obtained by merging combined maps with subtraction maps. Such merging consisted in averaging the maps, weighting by the accuracy of each of them:

$$\begin{array}{l} t_{Final}=w_{Between-groups}\cdot t_{Between-groups}\\ +w_{Patients-Controls}\cdot t_{Patients-Controls} \end{array}$$

The weight of a combined map ranged from 1 at peaks to 0 in voxels far from any peak. Similarly, the weight of a subtraction map ranged from 1 at peaks found in both patient and control maps to 0 in voxels far from any patient or control map peak. More specifically, weights were calculated as follows: (a) SDM pre-processing was carried out with all peaks set to 1 to derive the degree of accuracy of each map, (b) averaging of patient and control maps of accuracy was carried out to derive subtraction accuracy maps, and (c) scaling of the combined and subtraction accuracy maps was carried out in order that they sum to unity.

Finally, the effect-size and the effect-size variance maps of all studies were introduced into a meta-analytical random-effects model, which takes intra-study variability, sample-size, and between-study heterogeneity into account. Assessment of statistical significance was based on a distribution-free permutation test (Radua et al., 2011).

### Multimodal meta-analysis of cognitive difficulty and time perception

We performed a multimodal meta-analysis to combine the findings from the above-described SDM meta-analysis of studies comparing two levels of cognitive difficulty with those from an ALE meta-analysis on three neuroimaging studies exploring time perception in schizophrenia (see Supplementary Material, Table 1). This latter was previously published by our team (Ortuño et al., 2011).

The aim of this multimodal analysis was to detect brain regions that are activated or deactivated by both cognitive difficulty and time perception tasks. We, therefore, overlapped the map of the BOLD response to cognitive difficulty with the map of the BOLD response to time perception. This was conducted using a modification of the probability of the union of the maps (Radua et al., 2013), rather than a simple overlap of them, as the former has been shown to deal with the presence of error in the *p*-values of the individual meta-analysis. The combination of the ALE and the SDM meta-analysis was then computed as the union of their probabilities (Radua and Mataix-Cols, 2012). Final results were thresholded with voxel *p* < 0.01, peak *p* < 0.001, and cluster extent >10 voxels.

## Results

The search strategy identified 1134 citations. Duplicated papers were removed. From the remaining studies 1091 were excluded because they did not meet the eligibility criteria. A total of 43 studies were included in the meta-analysis. Of these, 14 involve a standardized cognitive task such as N-back, Sternberg, Stroop, or Continuous Performance Test. Basic cognition (such as executive functions, working memory, attention or verbal fluency) is examined in 24 papers; social cognition, in 11; and controlled processes, in the remaining eight studies. Sample size for the included studies ranges from a minimum of six participants for each group to a maximum of 118, with a total participation of 954 schizophrenia patients and 999 healthy volunteers (Table 1).

**Figure 1:**
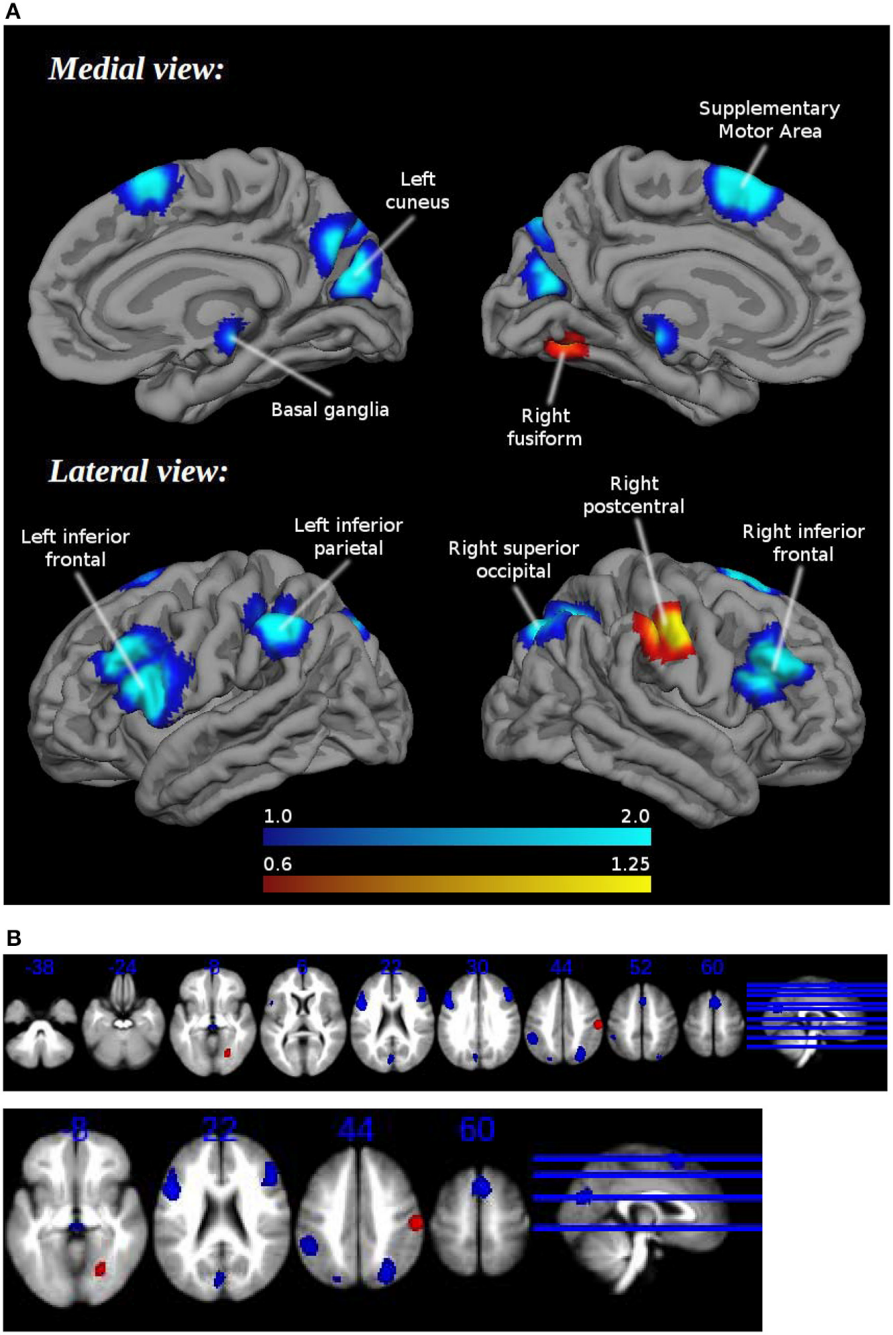
**(A)** Brain regions engaged during tasks requiring cognitive control: differences between healthy controls and schizophrenia patients. **(B)** Brain regions engaged during tasks requiring cognitive control: differences between healthy controls and schizophrenia patients. Multislice.

Patients showed hypoactivation in bilateral inferior frontal and superior occipital gyri, right supplementary motor area, left inferior parietal gyri, left cuneus, and red nucleus. Patients also exhibited hyperactivation or failure of deactivation in right postcentral and fusiform gyri (Figure 1, Table 2).

**Table 2:** **Brain regions engaged during tasks requiring cognitive control: differences between healthy controls and schizophrenia patients**.

**Location**	**Peak**			**Cluster**	
	**MNI**	**Z**	**P**	**Voxels**	**Breakdown**
Right postcentral gyrus, BA 3	60, −18, 42	1.251	0.000029802	279	Right postcentral gyrus (200), mostly BA 3
					Right supramarginal gyrus (71), mostly BA 3/1
Right fusiform gyrus, BA 19	26, −66, −8	1.005	0.000230074	127	Right inferior network, inferior longitudinal fasciculus (52)
					Right fusiform gyrus (44), mostly BA 19
					Right lingual gyrus (26), mostly BA 18
Left inferior frontal gyrus, opercular part	−46, 10, 26	−2.041	0.000001013	927	Left inferior frontal gyrus, opercular part (365), mostly BA 44
					Left inferior frontal gyrus, triangular part (318), mostly BA 48
					Left precentral gyrus (109), mostly BA 44
					Left middle frontal gyrus (61), mostly BA 44
					Left frontal aslant tract (35)
					Left frontal inferior longitudinal fasciculus (16)
					Corpus callosum (12)
					Left superior longitudinal fasciculus III (10)
Right inferior frontal gyrus, triangular part, BA 44	48, 26, 28	−1.955	0.000001729	642	Right inferior frontal gyrus, triangular part (331), mostly BA 45
					Right middle frontal gyrus (215), mostly BA 45
					Right inferior frontal gyrus, opercular part (68), mostly BA 44
					Right frontal inferior longitudinal fasciculus (25)
Right superior occipital gyrus, BA 7	28, −68, 44	−1.536	0.000065565	474	Right superior occipital gyrus (192), mostly BA 7
					Right angular gyrus (99), mostly BA 7
					Right superior parietal gyrus (98), mostly BA 7
					Corpus callosum (47)
					Right superior longitudinal fasciculus II (12)
Right supplementary motor area, BA 6	4, 14, 58	−1.710	0.000013709	462	Right supplementary motor area (259), mostly BA 6
					Left supplementary motor area (196), mostly BA 6
Left inferior parietal (excluding supramarginal and angular) gyri, BA 40	−50, −42, 46	−1.392	0.000229657	351	Left inferior parietal (excluding supramarginal and angular) gyri (333), mostly BA 40
					Left postcentral gyrus (11), mostly BA 2
Left cuneus cortex, BA 18	−4, −76, 24	−1.210	0.000986218	277	Left cuneus cortex (153), mostly BA 18
					Left precuneus (39), mostly BA 7
					Left calcarine fissure/surrounding cortex (37), mostly BA 18
					Corpus callosum (18)
					Left median network, cingulum (15)
Basal ganglia	4, −26, −6	−1.078	0.002775669	57	
Left superior occipital gyrus, BA 19	−20, −76, 42	−1.179	0.001261711	53	Left superior parietal gyrus (31), mostly BA 7
					Left superior occipital gyrus (22), mostly BA 19

*Threshold: voxel P < 0.00500, peak SDM-Z > 1.000, cluster extent size ≥10 voxels. Breakdown regions with < 10 voxels are not reported*.

Jacknife analysis showed that differences between groups in bilateral inferior frontal gyri, right superior occipital gyrus, the right supplementary motor area, and the red nucleus were found in all combinations of studies, indicating a high replicability. Between-group differences in the right postcentral gyrus, the right fusiform gyrus, the left inferior parietal gyrus, the left cuneus, and the left superior occipital gyrus failed to appear in some combinations of studies.

Visual inspection of peak funnel plots did not reveal potential publication bias or other gross abnormalities. The Egger test was only marginally significant in the peak of the red nucleus (4, -26, -6; see Supplementary Material).

Findings are consistent with our team's previously published ALE meta-analysis on neuroimaging of time perception in schizophrenia. This previous work concluded that schizophrenic patients showed, in comparison to healthy controls, significantly lower activation of the right precentral gyrus [Brodmann Area (BA) 6], the superior (BA 9), and middle (BA 8 and 10) frontal gyrus, the left anterior cingulate (BA 32), the right parietal cortex (BA 39), the right putamen and the thalamus (see Supplementary Material; Figure 1, Table 2).

**Figure 2:**
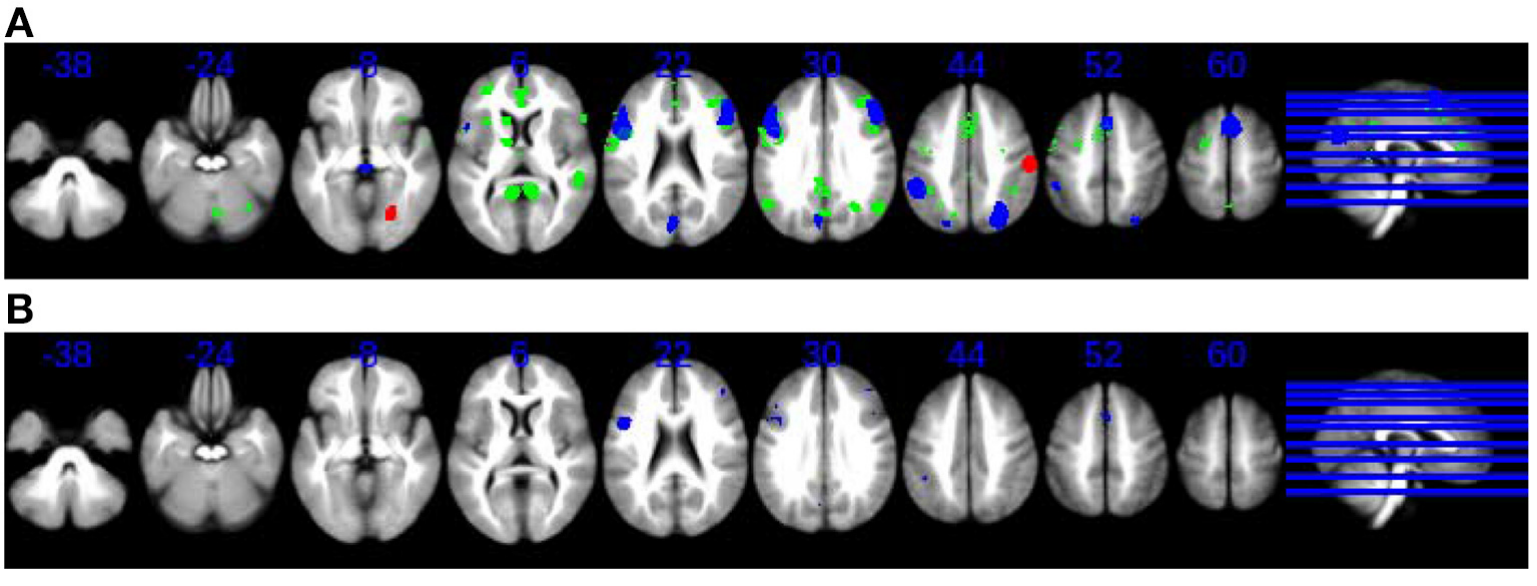
**(A)** Overlap and lack of overlap between brain regions engaged during time perception tasks and during tasks requiring cognitive control. Axial slices in neurological convention showing regions with statistically signification activation only during time perception tasks (SDM meta-analysis, green) and regions with statistically signification activation during tasks requiring cognitive control (SDM meta-analysis, blue, and red). Red for hyperactivations (patients > controls in difficult > easy) or for failures of deactivation (patients < controls in difficult < easy), and blue for hypoactivations (patients < controls in difficult > easy) or hyperdeactivations (patients > controls in difficult < easy). **(B)** Overlap and lack of overlap between brain regions engaged during time perception tasks and during tasks requiring cognitive control. Axial slices in neurological convention showing regions with statistically signification activation both during time perception tasks and during tasks requiring cognitive control (blue).

The results of the multimodal meta-analysis (Figure 2B) suggest bilateral overlapping of cortical and subcortical regions: particularly frontal areas (mainly right BA 6), as well as parietal regions and the basal ganglia. The participation of these regions, primarily in the right hemisphere, was reduced in schizophrenic patients relative to control subjects, not only by time perception tasks but also by an increase in the difficulty of non-temporal tasks.

Note that overlapping was only found in those brain regions that were deactivated or hypoactivated by cognitive difficulty. However, the brain regions, which were activated by cognitive difficulty, did not overlap with the map of the BOLD response to time perception.

Together with the overlapping cortical and subcortical regions during both task types, statistically significant activation was found to occur in a group of non-overlapping brain regions (Figure 2A): the right thalamus and the left anterior cingulate were specifically activated only in time perception tasks whereas, the bilateral superior occipital gyrus and the right fusiform gyrus were only activated during tasks requiring cognitive effort.

## Discussion

Overall, our findings support the hypothesis that timing structures are activated by an increase in the difficulty of non-temporal cognitive tasks in schizophrenia. The findings are in broad agreement with a recent meta-analysis of functional neuroimaging studies in healthy volunteers (Radua et al., 2014a). Both meta-analyses suggest a partial overlap of cortical and subcortical brain regions engaged in time perception tasks with regions engaged in tasks requiring increased cognitive effort. Specifically, we found a pattern of fronto-parietal and basal ganglia activation common to timing and increased cognitive effort. In schizophrenia patients, the involvement of most of these overlapping cortical and subcortical areas, primarily in the right hemisphere, was reduced in comparison to that in healthy controls.

The involvement in common of some regions by both timing and non-temporal cognitive tasks can be interpreted to indicate that these two functions require similar cognitive abilities. During cognitive tasks with various levels of effort or control, some temporal processing is engaged. Thus, we hypothesize that certain brain regions (such as the insula) traditionally associated with timing are engaged during non-temporal cognitive tasks in response to increases in the level of difficulty. Furthermore, since timing tasks involve different cognitive processes (such as sustained attention, working memory, decision making, or preparation of motor responses), specific brain regions usually associated with these domains (such as the prefrontal cortex and fronto-parietal regions) are hypothesized to be engaged during these tasks.

Another recent meta-analytic study (Niendam et al., 2012) found evidence of a superordinate cognitive control network subserving diverse executive functions. This network involves dorsolateral prefrontal, anterior cingulate, and parietal cortices. The results of our study support the idea that the aforementioned network exists, but they also suggest that the network responds to changes in task demands. With regard to the regions involved, the current meta-analysis coincided in large measure with other studies but indicated that the medial frontal (SMA), temporal insula, and basal ganglia should be included as part of what we propose functions as a *temporal-cognitive control network*.

To date there are only a few published neuroimaging studies of timing in schizophrenia (e.g., Volz et al., 2001; Ojeda et al., 2002; Ortuño et al., 2005; Davalos et al., 2011). We hypothesize, in line with previous theory laid out by Andreasen (1999) and in line with the findings discussed below, that the observed timing impairment displayed in schizophrenia is mediated by a specific fronto-thalamo-striatal dysfunction. A recent functional neuroimaging study (Davalos et al., 2011) that examined the effects of task-difficulty in temporal processing in schizophrenia patients compared to healthy controls found, as we do here, that neuroanatomical regions known to be engaged in timing (SMA, the insula/operculum and striatum) showed signs of dysfunctionality in schizophrenia patients. The higher the level of task difficulty, the greater were found to be the differences in engagement of these regions between patients and controls. These findings, however, are not inconsistent with those of a fMRI study of an exclusively healthy population (Tregellas et al., 2006): the authors concluded that activation of certain regions (including SMA, insula/operculum and the striatum) during timing tasks is load-dependent.

What role does the SMA play in dysfunctional temporal processing in schizophrenia? The SMA has been proposed as a key structure during timing (Rao et al., 2001; Macar et al., 2002; Ferrandez et al., 2003; Tregellas et al., 2006) in the “pulse accumulation” process (Macar et al., 2004), and in attending to an internal timeline against which timing comparisons can be made (Coull et al., 2004). Whilst the role of the SMA is traditionally seen to be purely motor-oriented, a recent review considers that it may be activated by demand for implementation of several cognitive tasks: mental arithmetic, spatial and non-spatial working memory, attention control, silent work production, and conceptual reasoning (Hanakawa et al., 2008). The implication of a dysfunctional SMA is consistent with the idea proposed by Rao et al. (2001) of an early cortical failure related to attention disturbances leading to temporal processing deficits in schizophrenia.

In agreement with the Radua et al. meta-analysis (Radua et al., 2014a), the current meta-analysis found the occipital cortex (BA 19) to be a region engaged by tasks requiring cognitive effort. This suggests that this region together with the claustrum is engaged not only in time perception but also in executive functioning.

Since the participation of most of the cortical and subcortical regions primarily in the right hemisphere is reduced relative to healthy subjects, this finding suggests that a pattern of disconnectivity of the timing circuit is a characteristic of the schizophrenia condition (Ortuño et al., 2011).

Owing to the wide overlapping between neural networks involved in high-level cognitive functions and temporal processing, timing performance could be a sensitive measure of cognitive functioning and a reliable indicator of impairment to the underlying neural substrate (Piras et al., 2014). In fact, temporal processing has been suggested as a “cognitive primitive,” a fundamental neuropsychological process that has a broad influence on cognition (Fuster et al., 2013).

The common networks that support modulation of effort during non-temporal cognitive tasks also support timing tasks. This finding somewhat belatedly provides backing to Aristotle's philosophical concepts that timing is related to the perception of change and that time is ubiquitous. As time is omnipresent in the processes of nature, so must time be dealt with by all the higher human cognitive functions (Ortuño and Alústiza, 2014).

It should be noted that the studies we selected for our meta-analysis compared neural activation between two levels of difficulty of their respective experimental tasks. These studies, therefore, reflect how the brain responds to an increase in cognitive load, an increase in the effort required, or an increase in the intensity of what is demanded while the underlying nature of the cognitive function of the task remains essentially the same. The fact that all the studies involve this kind of change in cognitive effort is fundamental to the design of the study and, we believe, critical to the interpretation of our results.

Impaired performance in tests sensitive to different functions (involving the frontal, temporal, hippocampal, parietal, striatal, and cerebellar areas) delineates schizophrenia. In this disease, therefore, there is evidence of a generalized cognitive deficit affecting general neurobiological mechanisms (Gómez et al., 2014). While neuroscience studies indicate that timing-related symptoms are only primary to cognitive impairments and secondary to thought disorders, psychopathological and phenomenological studies strongly imply that disturbance in time perception is the core symptom in schizophrenia.

Difficulty in controlling the involvement of other cognitive domains in temporal processing execution contributes to the continued debate over the specificity of timing dysfunction. The question is whether the dysfunction is associated with a disturbance in central temporal processes or whether it is attributable to a cognitive or biological dysfunction (Bonnot et al., 2011). It should be noted that the involvement of cognitive brain areas in the discrimination of short (50–500 ms) durations and with automated (pre-conscious) processes is less than that in the discrimination of longer durations and conscious processes.

Three conclusions can be drawn from this study. First, in schizophrenia, there is a widespread network of brain regions (frontal, parietal, and basal ganglia) engaged both in timing tasks and in tasks involving an increase in the cognitive effort demanded for execution of non time-related mental processes. Second, these cerebral circuits, which might be called a *temporal*-*cognitive control network*, sustain and are common to all mental processes and operations that involve increases (and possibly also decreases) in cognitive load. Lastly, response deficits in this network are highly load-dependent, which suggests that generalized timing deficits in schizophrenia may involve a broad network dysfunction. An important implication of our findings is that the link between a dysfunctional timing network and other impaired cognitive functions only becomes evident when there is comparison of a task performed at different levels of cognitive effort.

A focus on the processing of temporal information offers a way to understand the cognitive deficits of schizophrenia and how these deficits might contribute to a variety of psychiatric symptoms and have an adverse effect on the everyday activities of patients. In this sense, we suggest that a deficit in timing be tentatively considered as a trait marker of the schizophrenia cognitive profile.

Inferences about the dysfunctional overlap observed in the present study are limited by the lack of a way to make an objective assessment of the supposed internal clock. This difficulty has led to dependence in our study on tasks involving both a temporal component and other non time-specific cognitive domains.

It should be noted that the network overlap might be due to a task difficulty effect on neural activation in the time perception studies included.

A methodological note: as far as we know, this study is the first to use the technique of multi-source pre-processing. It is through this technique that the main value of the meta-analysis is established. The main implication thus derived is that, in schizophrenia, the link between a dysfunctional timing network and other impaired functions becomes evident with an increase in the demand for cognitive effort.

It would be interesting to examine whether *temporal*-*cognitive control network* regions can be attributed to specific cognitive domains accessed by different tasks. Additionally, future research could address the questions of whether timing distortions are a manifestation of, or a mechanism for, cognitive and behavioral symptoms, and whether the relationship applies not only in schizophrenia but also in psychosis in general.

## Author contributions

Each co-author contributed substantially to the manuscript, addressing different tasks. IA contributed to the conception and design of the work, as well as to the acquisition and interpretation of data and drafting the paper. JR and AA contributed to the design of the work, data analysis and interpretation, and drafting and critical revision of the text in terms of intellectual content. MD contributed mainly in the acquisition of data for the work. FO contributed principally in the conception and design of the study. FO and EA gave final approval of the version to be published and agree to be accountable for all aspects of the work and to ensure that any questions related to the accuracy or integrity of any part of the work are appropriately investigated and resolved.

## Funding

Clínica Universidad de Navarra, Pamplona, Navarra, Spain. Instituto de Investigación Sanitaria de Navarra, Navarra, Spain.

### Conflict of interest statement

The authors declare that the research was conducted in the absence of any commercial or financial relationships that could be construed as a potential conflict of interest.
