# Supplementary material for: Meta-Analysis of Functional Neuroimaging and Cognitive Control Studies in Schizophrenia: Preliminary Elucidation of a Core Dysfunctional Timing Network
Source: Front Psychol. 2016 Feb 17;7:192. doi: 10.3389/fpsyg.2016.00192 (PMC4756542; doi:10.3389/fpsyg.2016.00192)
Supplement: Supplementary file 1 [file DataSheet1.PDF]

Supplementary Material

Meta-analysis of functional neuroimaging and cognitive control studies in schizophrenia: Towards elucidating a core dysfunctional timing network

Irene Alústiza*, Joaquim Radua, Anton Albajes-Eizagirre, Manuel Domínguez, Enrique Aubá, Felipe Ortuño

*** Correspondence:** Corresponding Author: ilalustiza@unav.es

1. Supplementary Data

Funnel Plots

Left inferior frontal gyrus, opercular part

Egger test

Bias: -0.03, t: -0.07, df: 41, p: 0.947

Funnel plot

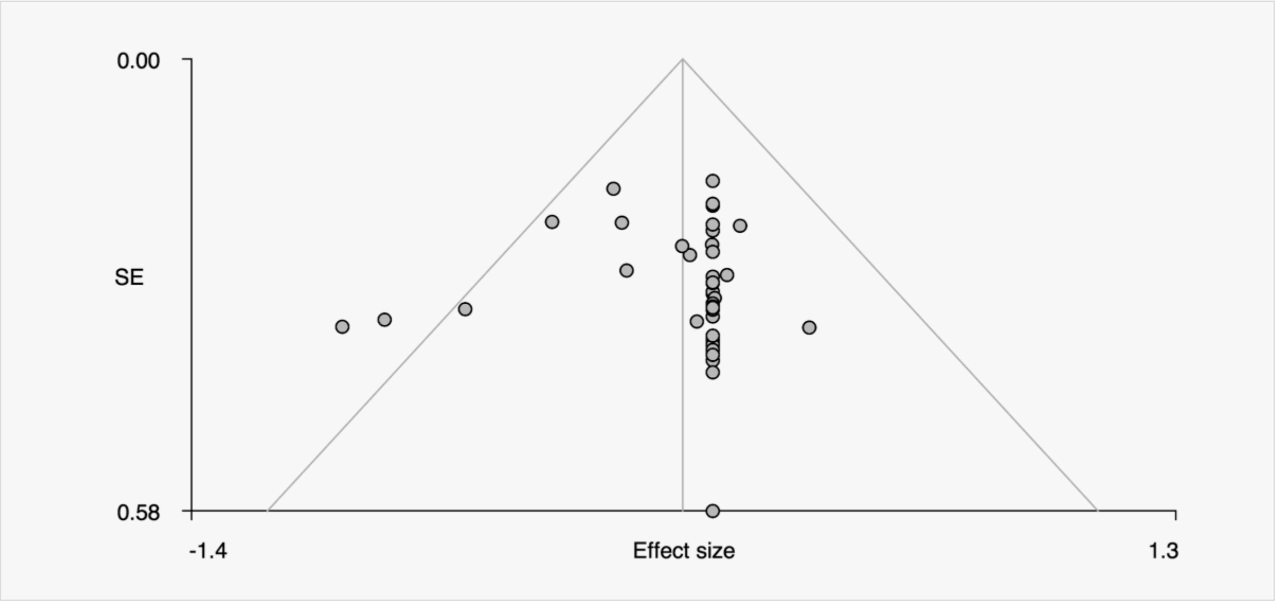

Right inferior frontal gyrus, triangular part, BA 44

Egger test

Bias: -0.35, t: -0.80, df: 41, p: 0.426

Funnel plot

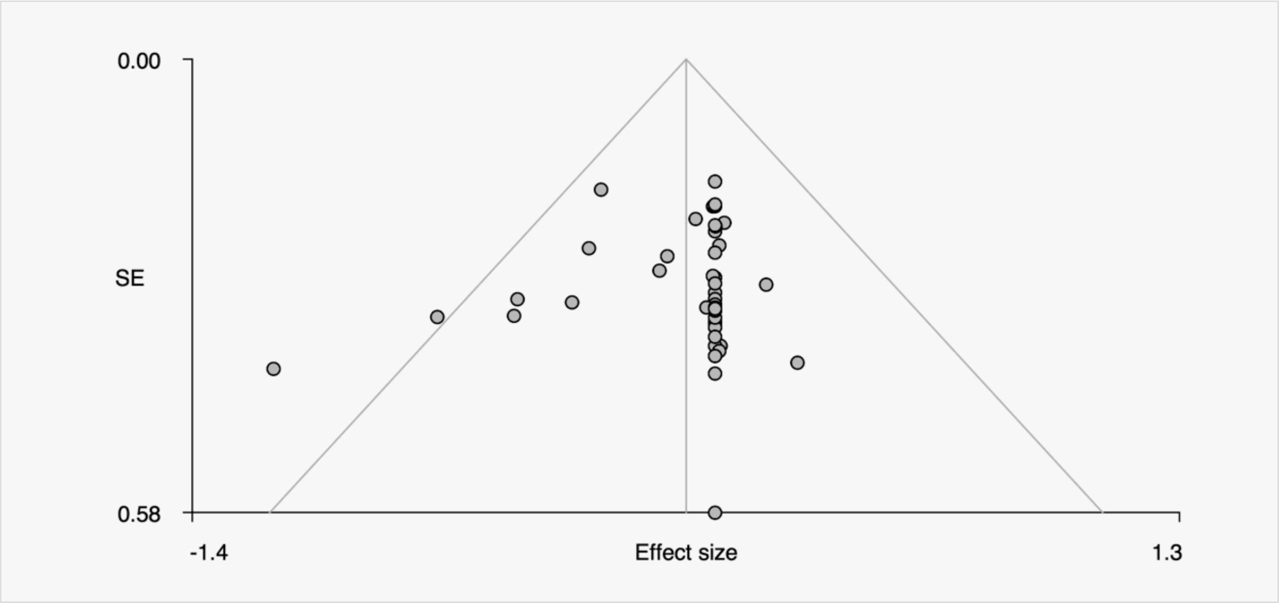

Right superior occipital gyrus, BA 7

Egger test

Bias: -0.06, t: -0.18, df: 41, p: 0.857

Funnel plot

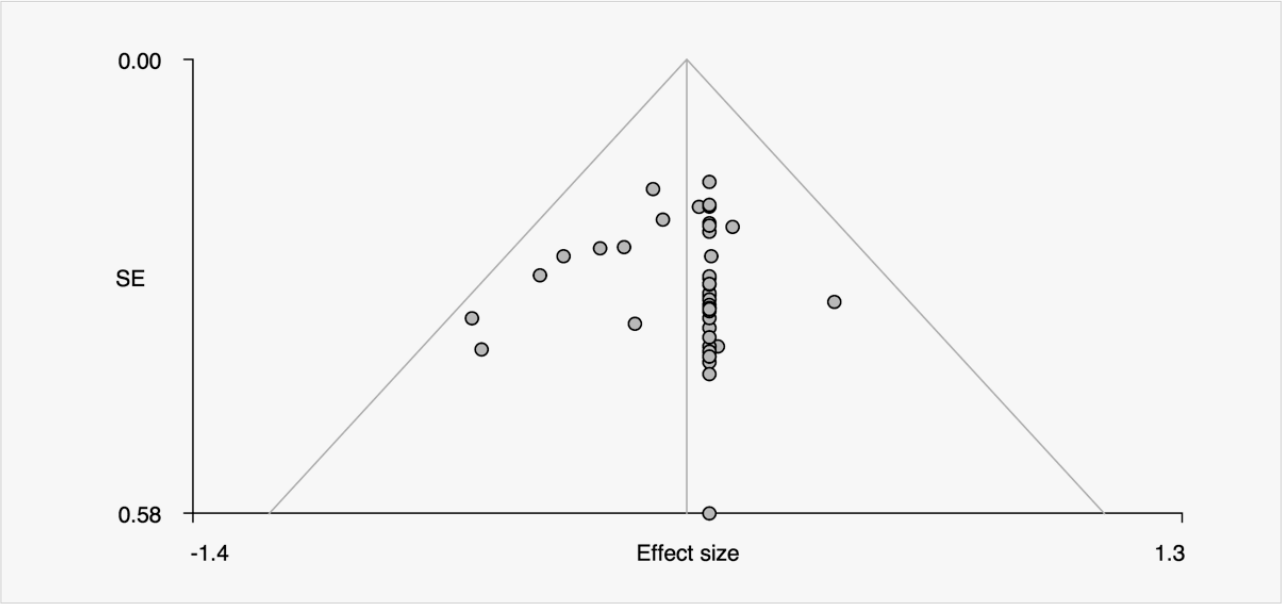

Right supplementary motor area, BA 6

Egger test

Bias: 0.11, t: 0.33, df: 41, p: 0.746

Funnel plot

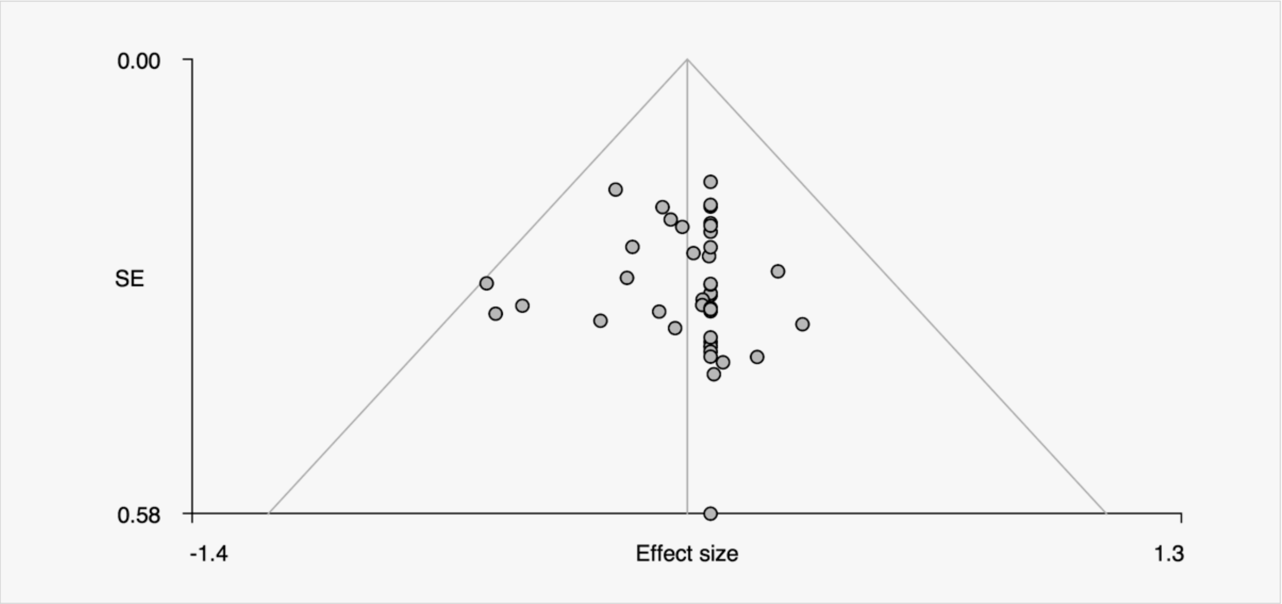

Left inferior parietal (excluding supramarginal and angular) gyri, BA 40

Egger test

Bias: -0.17, t: -0.37, df: 41, p: 0.714

Funnel plot

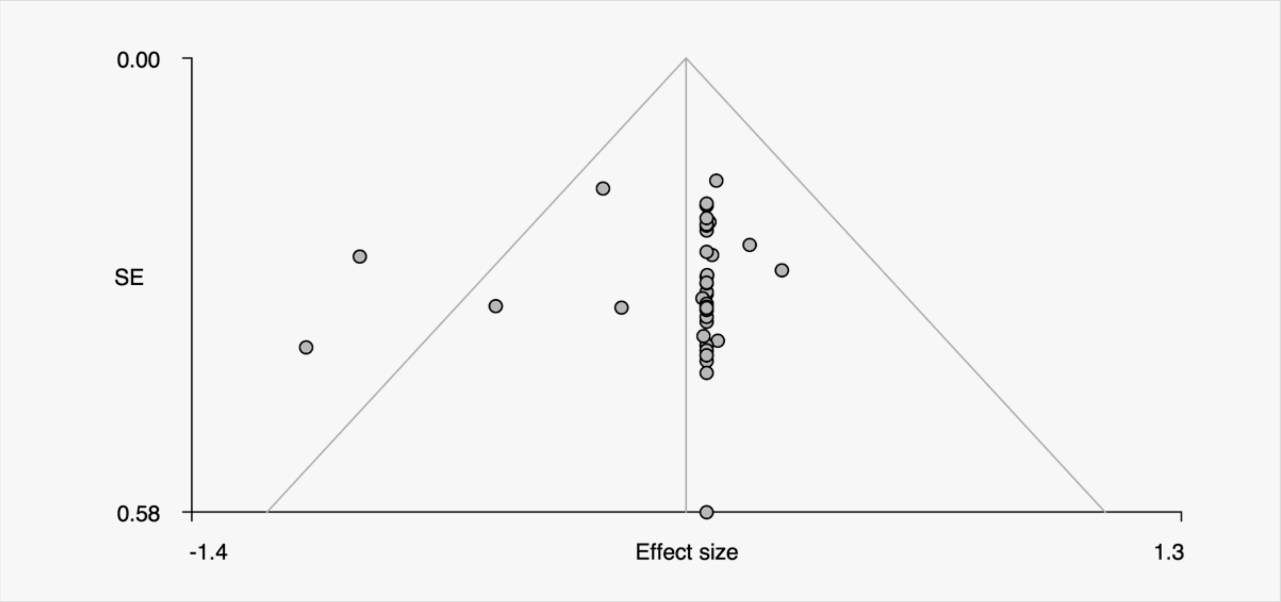

Left cuneus cortex, BA 18

Egger test

Bias: -0.39, t: -1.24, df: 41, p: 0.222

Funnel plot

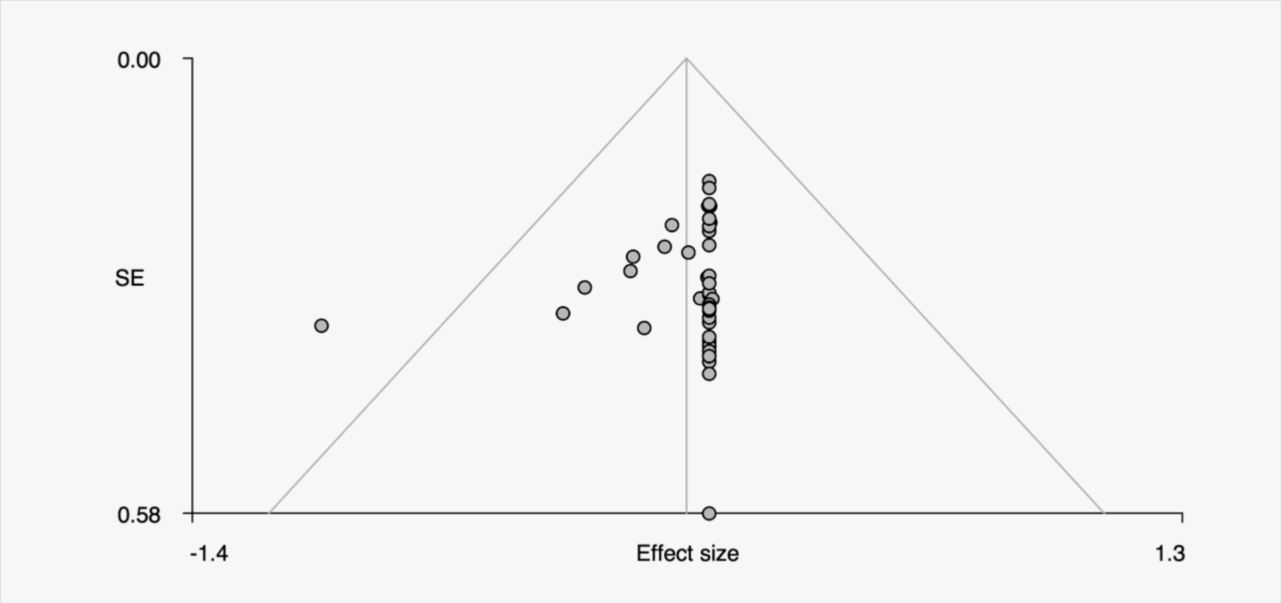

Basal Ganglia

<u>Egger test</u>

Bias: -0.66, t: -2.13, df: 41, p: 0.039

<u>Funnel plot</u>

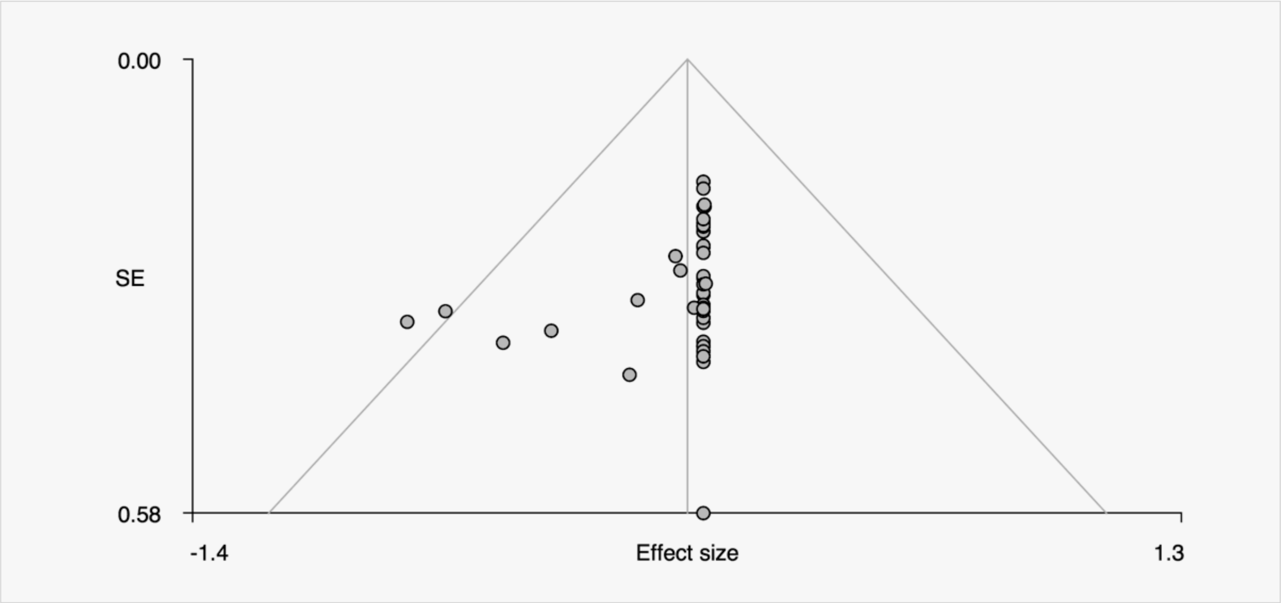

Left superior occipital gyrus, BA 19

<u>Egger test</u>

Bias: -0.31, t: -0.93, df: 41, p: 0.359

<u>Funnel plot</u>

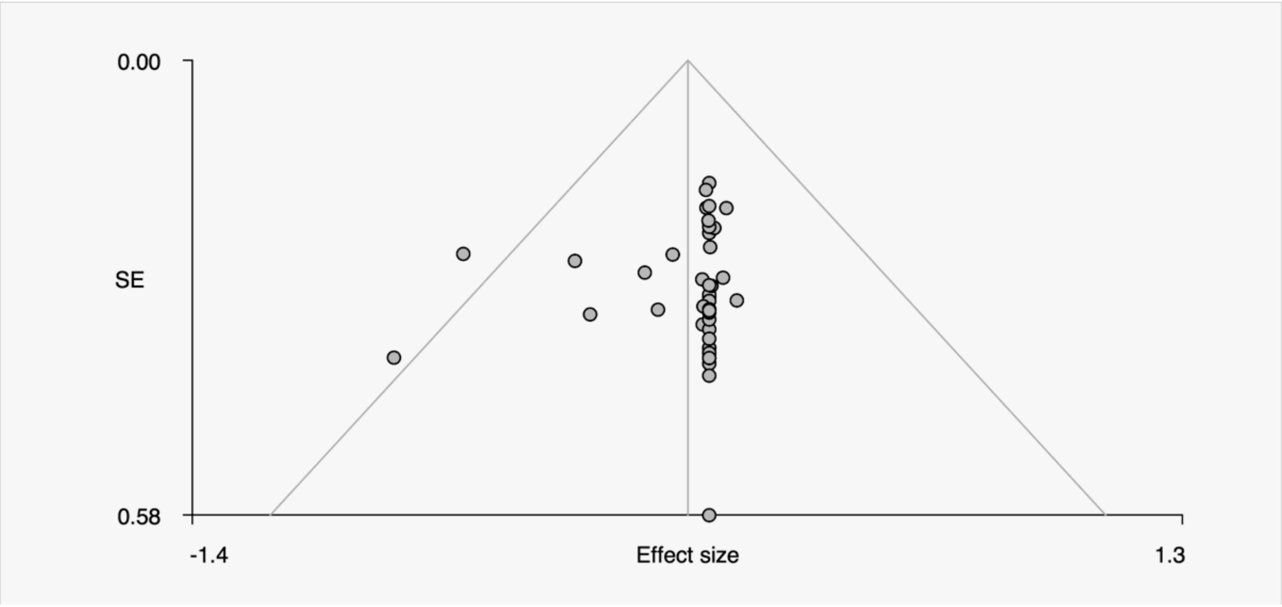

Right postcentral gyrus, BA 3

Egger test

Bias: -0.67, t: -1.90, df: 41, p: 0.065

Funnel plot

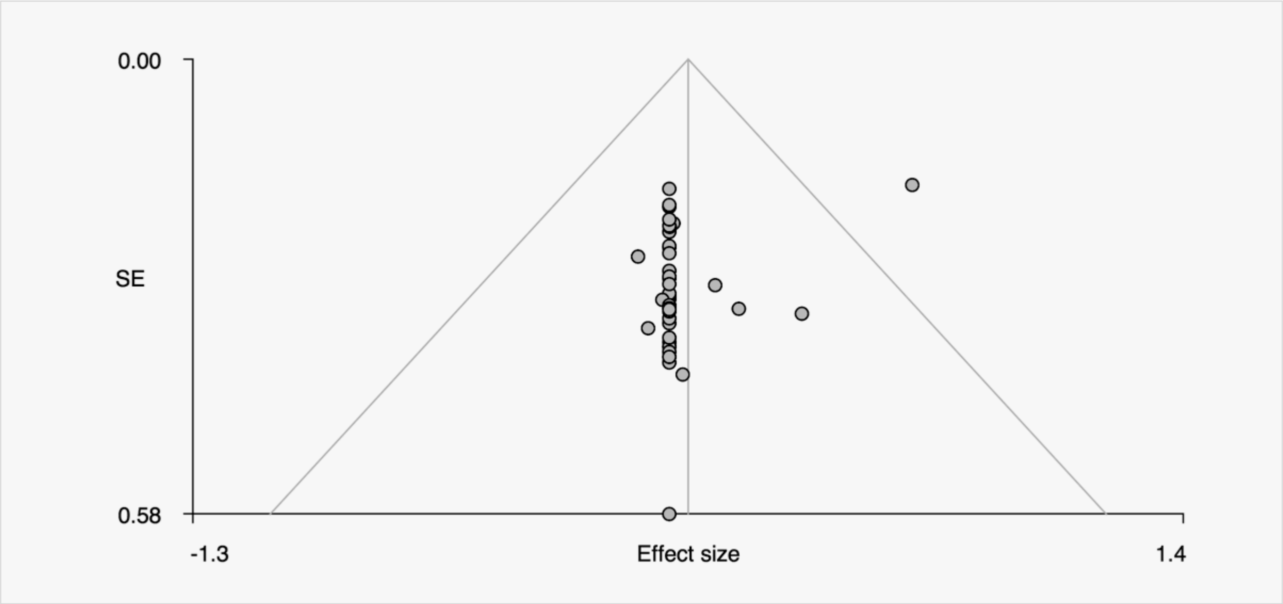

Right fusiform gyrus, BA 19

Egger test

Bias: -0.08, t: -0.19, df: 41, p: 0.848

Funnel plot

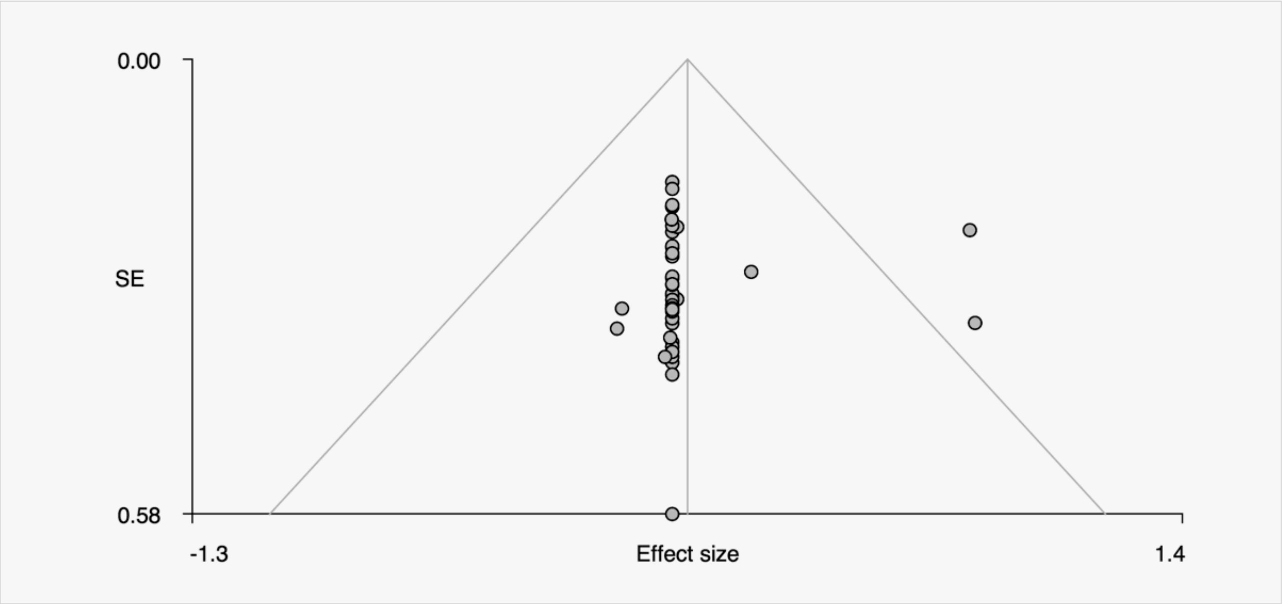

2. Supplementary Tables and Figures

2.1 Supplementary Tables

Supplementary Table 1
Studies of temporal processing included in ALE meta-analysis (Ortuño et al., 2011).

Author	Sample	Task	Included contrast
1. Volz et al., 2001	8 SZ<br>15 HC	Auditory discrimination task	Time estimation > Frequency discrimination
2. Ojeda et al., 2002	11 SZ<br>10 HC	Counting task	Mentally and silence counting > Auditory clicks rhythm counting
3. Ortuño et al., 2005	11 SZ<br>10 HC	Counting task	Mentally and silence counting > Auditory clicks rhythm counting

Note: SZ, schizophrenic patients; HC, healthy controls.

Supplementary Table 2
Significant activation likelihood clusters for time estimation tasks: differences between healthy controls and schizophrenia patients (Ortuño et al., 2011).

Location	Volume mm3	x	y	z	ALE
Lentiform nucleus (R)	1408	28	-14	0.89	0.007
Precentral gyrus. BA 6 (R)	1208	48	-6	5.66	0.008
Superior frontal gyrus. BA 9 (R)	560	16	38	34	0.006
Parietal lobe. Precuneus. BA 39 (R)	504	46	-70	34	0.007
Thalamus (R)	488	8	-7	11	0.006
Cingulate gyrus. BA 32 (L)	392	-9	24	28	0.007
Middle frontal gyrus. BA 8 (R)	224	36	36	40	0.006
Superior frontal gyrus. BA 10 (R)	128	14	66	8	0.005

2.2 Supplementary Figures

Supplementary Figure 1
ALE maps of time estimation studies: schizophrenia < healthy subjects (Ortuño et al., 2011).

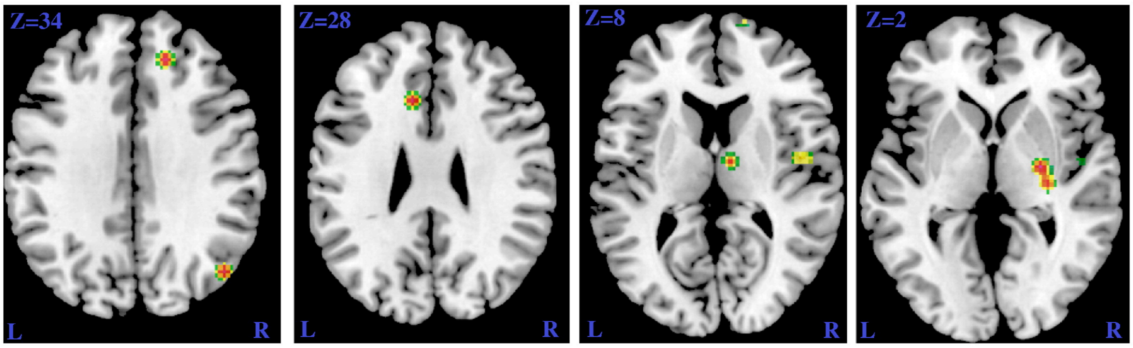

Superior frontal gyrus Inferior parietal lobe | Cingular Gyrus | Thalamus Insula | Pallidus-Putamen
